# Ethical issues in the clinical use of diagnostic technologies for rare causes of psychosis

**DOI:** 10.1192/bjp.2026.10675

**Published:** 2026-06-16

**Authors:** Matthew L. Baum, Graham Blackman, Adam Al-Diwani, Adam Handel, Dominique Endres, Sophie Binks, Jonathan Pugh, John Ward, Julian Savulescu, Ilina Singh, Belinda Lennox

**Affiliations:** 1 Department of Psychiatry, Brigham and Women’s Hospital, Boston, MA, USA; 2 Department of Psychiatry & Center for Bioethics, Harvard Medical School, Boston, MA, USA; 3 Department of Psychiatry, University of Oxford, Oxford, UK; 4 National Institute for Health Research (NIHR) Oxford Health Biomedical Research Centre, University of Oxford, Oxford, UK; 5 Department of Neurology, University of Oxford, Oxford, UK; 6 Department of Psychiatry and Psychotherapy, Medical Center - University of Freiburg, Faculty of Medicine, University of Freiburg, Freiburg, Germany; 7 Uehiro Oxford Institute, University of Oxford, Oxford, UK; 8 Faculty of Philosophy, University of Oxford, Oxford, UK; 9 Department of Psychiatry, University of Cambridge, Cambridge, UK; 10 Centre for Biomedical Ethics, Yong Loo Lin School of Medicine, National University of Singapore, Singapore, Singapore

## Abstract

An estimated 5% of cases of first episode psychosis are due to rare and sometimes reversible causes that can be identified though diagnostic investigations that are currently less common in psychiatry, such as lumbar puncture, specialized brain imaging, or genetic testing. The current clinical practice of the use of such technologies, however, raises several challenging ethical questions. Drawing on our multi-disciplinary perspective spanning psychiatry, neurology, philosophy, neuroscience, sociology, and medical ethics from three countries we identify emerging ethical issues in the diagnosis of rare causes of psychosis. We group challenges in three main categories 1) diagnostic justice, 2) moral responsibility, and 3) unintended consequences of diagnostic workup. Justice challenges surrounded role of a) “luck”, b) social capital, and c) prior psychiatric diagnoses in determining access to workup. Moral responsibility challenges surround the extent to which a) clinicians should be expected to know the red flags and workup for rare or ultra-rare causes, b) those who run health systems should enable knowledgeable clinicians to provide the requisite workup. Challenges related to unintended consequences include the risk of pursuing workup of rare causes to reinforce a) psychiatric exceptionalism, b) paternalistic decision making that does not allow space for patient preferences. Finally, we reflect on unresolved issues and future directions.

## Introduction

New onset psychosis is a psychiatric presentation requiring careful clinical judgment to determine the underlying etiology and to ensure acute stabilization. Out of 100 people, 3 to 4 are estimated to experience psychosis over the course of their lifetime^1^. Of these, an approximate 5% will experience psychosis secondary to an identifiable, rare underlying cause (which are sometimes called “secondary causes”), including autoimmune brain disease^2^. The results of successfully finding potentially treatable autoimmune causes can be drastic, sometimes leading to rapid reversal of the presenting symptoms. The consequences of identifying other secondary diagnoses, such as infectious, metabolic, oncologic, are similarly dramatic. However, the process of attempting to detect which individuals with new onset psychosis have an identifiable (and potentially reversible) cause is not at all straightforward, requires the use of advanced or sometimes invasive diagnostics with which many psychiatrists may have limited knowledge or experience, and raises a number of under-discussed ethical challenges. However, there is not a large evidence base or literature on these ethical issues to which clinicians might appeal for guidance. For illustrative purposes, for example, if a clinician were recently to search PubMed for two common terms for rare identifiable causes of psychosis and ethics/ethical, this would have yielded zero results (see Box 1). While an expanded search would yield relevant clinical guidelines^3^, or articles concerning ethical issues of other diagnostic categories^4^ or hypotheses^5^, there is little directly on ethical issues in the diagnostic workup of rare causes of psychosis. In the absence of such literature, therefore, we bring together in this feature our multidisciplinary perspectives spanning psychiatry, neurology, philosophy, neuroscience, sociology, and medical ethics from 3 countries to begin to identify emerging ethical issues in the diagnosis of identifiable causes of psychosis. Such a highly interdisciplinary perspective has proven useful for discussions of neuroethics in other emerging areas^6^, and we hope will catalyze dialogue here, including towards what sort of future studies are needed

A core feature that emerges from cases like this are ethical issues related to justice, including equity of access to timely and appropriate workups. Other categories of ethical challenges surround a) individual responsibilities, e.g. the degree to which physicians should be expected to know about when, and how to suspect rare etiologies, especially given the risks of premature closure of diagnostic evaluation when knowledge is partial, or the degree to which institutions should guarantee access to diagnostic technologies and ensure care pathways that can support diagnosis of rare etiologies, b) professional autonomy concerns, including who should decide that a rare but identifiable cause is too rare to workup, c) unintended harm concerns, e.g. how workup for an identifiable cause of psychosis can sometimes unintentionally lead to delays in diagnosis of primary psychiatric conditions and the treatment of psychiatric symptoms with conventional approaches, or unintentionally reinforce stigma related to the potential of a primary mental illness, and d) how to weigh the risks and benefits of pursuing extensive investigation, including the risk of iatrogenic harm, both of the investigation itself (e.g. cost, post-LP headache) and unintended harm concerns, e.g. as described in recent commentary^7^.

We expand upon each of these ethical issues in the discussion that follows. Our tripartite framing of ethical challenges was chosen through critical dialogue amongst the manuscript authors, drawing upon common theory in philosophical bioethics; we acknowledge that there are some themes that cut across these categories.

## Ethical challenges

### Justice

Concerns broadly related to healthcare justice and equity cut across many clinical cases in the workup of rare causes of psychosis. Especially salient are concerns over barriers to accessing the relevant diagnostic workups, and that access can often be determined by “luck” of *where* a patient presents rather than core features of their presentation (e.g. is highly susceptible to variability in how hospitals, healthcare systems were organized) or by factors that could reinforce existing inequities in the care of people with severe psychiatric symptoms.

#### Role of “luck” in determining access to workup

A striking feature, in our observation, is the degree to which “geographic serendipity” is felt to play a role in many of the cases. For example, consider a case of psychosis that was found to be due to early frontotemporal dementia due to a genetic cause (C9orf72) at a psychiatric hospital which happened to have made a strategic decision to recently hire a full-time consultant neurologist (when such a structure is uncommon at most stand-alone psychiatric hospitals)^8^; though the referral to the neurologist was made upon clinical suspicion, if that patient had happened to live in a different hospital catchment area and gone to another high-quality hospital that had not made this strategic decision, access to such a workup would have been much more limited and could have been much delayed. As another example, consider a case of autoimmune encephalitis that was identified because a hospital system had integrated early LP into its workflow (while such workflows are not present or even feasible at other hospitals of similar quality due to variability in the availability of LP for these indications). We also observe that in some health systems or countries, only specific versions of tests, with known limitations in sensitivity were available compared to more sensitive tests because of contracts between health systems and particular clinical pathology business entities. It is certainly fortunate that these patients happened to present in the right country, at the right hospital, to the right team, at the right time; it also certainly seems unfair (or at least unfortunate), that the nation, local geography, and idiosyncrasies of the organization of health systems and the extent to which providers are comfortable with invasive or advanced diagnostics should determine whether a patient gets a particular workup for identifiable causes, rather than features we would agree are clinically and morally more relevant.

#### Role of social capital in determining access

Just as they determine access to health services generally, social and other kinds of capital determine access to the right kind of workup for rare causes of psychosis. For example, consider cases where a patient’s family advocated for a particular workup that was integral in finding a rare cause of psychosis and may not otherwise be pursued. Whether a patient has an advocate at their bedside, and whether they have the knowledge to know what to ask for, and whether they feel empowered enough to advocate for it are almost certainly influenced by sociodemographic privilege. Moreover, in certain contexts, the availability of certain advanced diagnostics can be highly dependent on insurance coverage or ability to pay, further augmenting concern that workup for rare identifiable causes might be disproportionally affected by a patient’s social and financial resources. Similarly, at times, these factors may influence not only access to clinically reasonable tests, but the likelihood of the sort of unintended harms we discuss later in this paper.

#### Role of prior psychiatric diagnoses and stigma in determining access

The third type of inequity in access can be seen in clinical cases where elements of concerning presentations for a rare cause of psychosis were attributed to the existence of a prior psychiatric diagnosis, even when the connection with that diagnosis was spurious or weak. For example, a person in their 50s who had a previous diagnosis of a personality disorder and a history of suicide attempts but developed a subacute onset of delusions and auditory hallucinations. Though the likelihood of a new-onset primary psychotic disorder such as schizophrenia would be uncommon in this age group^9^, it was a working diagnosis for almost a year until re-evaluation by a new team that involved behavioral neurology, which identified fasciculation and focal neurological deficits prompting a series of advanced diagnostic tests including positron emission tomography (PET) of the brain and genetic testing which revealed a c9orf72 expansion consistent with a behavioral variant frontal temporal dementia. Others had seen similar cases where they wondered if inappropriate anchoring on a pre-existing neurodevelopmental or psychiatric diagnosis delayed the consideration of identifiable causes of the new symptoms and appropriate use of invasive or other diagnostic medical technologies. “Anchoring bias” is a well-known cognitive bias in which prior information, such as a diagnosis, has undue influence on subsequent clinical decision making^10^ (for example leading to diagnostic overshadowing), i.e. as if the thinker is physically anchored to one spot and has difficulty moving to think about the situation in a different way. We have also encountered cases that raised the question, also discussed in the literature^11^ of whether the magnitude of anchoring bias was exacerbated in part due to pre-existing biases related to a patient’s membership in a marginalized group (e.g. due to the prior diagnosis being related to a stigmatized psychiatric category rather than a general medical category).

### Moral Responsibility

#### Individual epistemic responsibilities

When we say someone is morally responsible for an action, we generally mean it is *appropriate* that the person is blamed or praised for it; whether it is *appropriate*, depends on the degree to which the person i) had control over their action [called a “control condition” in philosophical literature] and ii) had the relevant knowledge about their action, e.g. that it would be a right or wrong thing to do [called an epistemic, or knowledge, condition] (e.g. see^12^). This epistemic component, in our experience, may be particularly relevant to the workup of rare causes of psychosis, as it is often unclear whether an action made in absence of knowledge about a rare cause of psychosis should validly be excused (the cause is rare, afterall) or whether there was some degree of moral responsibility because the fact in question *should have been known*. When “not knowing” is exculpatory or culpable has long been the subject of debate in the moral and legal literature, but is often underspecified when it comes to novel biomarkers or uses of biomarkers^12^.

How should we think about the degree to which psychiatric, emergency medicine, and neurology teams should have knowledge of the workup and management of rare or ultra-rare causes of psychosis? For example, we have discussed several cases where it appeared that lack of knowledge of warning signs for some rare causes (see this recent review of one example^13^) and of the invasive or less-common diagnostics that were required led to incomplete workups and pre-mature diagnostic closure. For example, consider a case of new acute onset psychosis in a person who had a recent (resolved) HSV encephalitis, who had a thorough workup including LP to rule-out *infectious* encephalitis, but CSF auto-antibodies were not sent to rule out a *post-infectious auto-immune encephalitis*, an exceedingly rare entity but at the same time a recognized complication of recent HSV encephalitis that is known to specialists^14^. Though the possibility of post-infectious autoimmune encephalitis was on the minds of the discussion group - given the special expertise in autoimmune etiologies of psychosis - this knowledge is not widely possessed by general psychiatry teams, and the incidence of the phenomenon is much rarer than one in a million^14, 15^. Should this level of knowledge be expected generally? Should this level of knowledge be expected about each of the large number of additional known rare congenital or acquired conditions associated with psychosis that could be recognized if physicians received training on how to recognize them^16, 17^? For many psychiatric training programs, there is tremendous variance in how much exposure clinicians get to rare phenomenon such as this or diagnostic investigations for rare etiologies, such as LP, auto-antibody testing, PET imaging, genetic testing; indeed many psychiatrists may have insufficient knowledge of when and how to use some of these investigations. Sometimes the level of knowledge expected is also part of what determines the legal standard or “standard of care” for the workup of a patient’s presentation; recently, others have also highlighted uncertainty in what constitutes the “standard of care” for the workup of autoimmune neuropsychiatric illness^18^. While each individual rare cause is by definition rare, others have pointed out that 1 in 15 people are estimated to have some kind of knowable rare disease and thus psychiatrists may have responsibility for considering rare diseases in a differential diagnosis^19^; nonetheless, the authors continue, the demandingness of this obligation can be managed if psychiatrists are not expected to know the minutiae of the diagnosis of rare disorders, but to be aware of general commonalities shared between many rare disorders that should alert them of when to consult a specialist. How to define the scope of these responsibilities will be an important challenge.

#### Systems responsibilities

Even when individual providers do have knowledge of indications for such tests, there may be times when health systems have significant structural barriers to accessing the tests indicated. For example, consider stand-alone psychiatric hospitals where the capacities for LP, PET, genetic testing are unavailable, or do not have easy access to behavioral neurology consultation. Or there may be features of patients’ insurance or providers’ hospital systems that create strong financial disincentives (to the patient, the provider, or the health system) for any testing or subspecialty consultation out of the ordinary. Returning to the discussion of responsibility above, this systems limitation might violate the “control” condition in a way that would limit the extent to which the individual provider might be held appropriately accountable, but the clinician may experience distress by participation in a system they feel is not doing the right thing by the patient (a phenomenon sometimes called “moral injury”). However, it raises questions about the moral responsibility of those running the health systems; e.g. what are the responsibilities to address such logistical and financial barriers to the workup of identifiable causes of psychosis? In many systems, especially those that are publicly funded, access to tests is often limited based on cost-benefit analysis; however, few cost-effectiveness analyses for rare, sometimes reversible causes of psychosis have been reports, and there are some reports that argue that some testing would be cost-effective^20^.

### Unintended consequences of workup for identifiable causes of symptoms

#### Risk of reinforcing psychiatric exceptionalism

Psychiatric exceptionalism can be defined as a belief that psychiatric care or conditions are somehow fundamentally different than other medical care or health conditions, and thus should be separate. A generally unanticipated ethical challenge can also be uncovered in cases where the workup for secondary causes or the finding of a rare identifiable cause seems to unintentionally reinforce patient or provider bias against use of mental health law (e.g. involuntary hospitalization), psychotherapy, and psychopharmacology. For example, consider a case where a frontal tumor was found in the diagnostic workup of worsened emotional and behavioral disturbance in a man with a history of post-traumatic stress disorder (PTSD). When the tumor was found, it seemed to reinforce a form of ‘therapeutic nihilism’; therapeutic nihilism refers to a belief that efforts to treat a condition will be futile or harmful. In this case, it was assumed that psychiatric medications or cognitive behavioral therapy (CBT) would be unhelpful (even though these approaches were subsequently found to be therapeutically beneficial). Or cases where a patient had tremendous anxiety and paresthesias in the setting of an autoimmune disease and was hesitant to try anti-anxiety medications as they did not want to mask the “cause of the problem”. Or finally, a family member who was angry that the mental health act had been used initially for involuntary detention when the patient’s symptoms ended up being due to a rare identifiable general medical condition. Could this family member rightly blame the health system even though, at the time, on the balance of probabilities, it would have been statistically more likely that there was a primary psychiatric cause to the symptoms, and detainment under the mental health act might reasonably have enabled access to a clinical team with more experience in managing behavioral risks safely? Ultimately, these tensions often appear to reflect implicit beliefs that mental health care and general health care should be mutually exclusive; on the other hand, the sort of uncommonly fluid crisscrossing of the longstanding divide between mental health care and general health care encountered in these cases could provide a unique opportunity for dialogue that promotes a more unified interdisciplinary approach to healthcare.

#### Best interests of patients and risk of false-positives

We also observe that these types of cases often stimulate vigorous discussion of the precise pre-test probabilities of various identifiable causes as a determining factor for whether testing should be offered. Of course, accurate testing can clearly benefit patients by illuminating a treatment pathway; this is especially so for when the test concerns a rare identifiable cause that is likely reversible if caught early. Nonetheless, no test is completely accurate, and there are several potential harms associated with the tests considered in this context. These include, for example, iatrogenic risks (e.g. post-LP headaches, anxiety around incidentalomas), as well as the harms associated with false positives^21^, including unnecessary medical interventions and psychological distress, the need for further testing or the extended delay of initiation of standard of care psychiatric treatments (e.g. if a patient, family, or team advocated exhaustive diagnostic evaluation before initiating psychiatric treatments). ^22^Indeed, there is an increasing acknowledgment that patients can be harmed by over-investigation^23^, and there is also a need to be wary of the prospect that extensive testing can foment stigma. There is therefore a significant trade-off between the potential harms and benefits of testing in this context^24^. For example, one group ^22^ reported a case where false-positive CSF anti-NMDAR antibodies (hypothesized to be from blood contamination of CSF from a traumatic LP) led to extensive further testing, use of IVIg (a costly and limited resource) and marked psychiatric decompensation before a mood stabilizer was eventually trialed to good effect.

How should such trade-off be managed? Some suggest that, because of the possibility of such false-positives, testing should be restricted to only patients with high pre-test probability of true positivity^22^. Yet the frequencies of false positives are often poorly defined, and the magnitude of the pre-test probabilities that would morally justify offering testing (or not offering testing) in need of clarification. Rigorous risk analysis to quantify the frequency and consequences of misdiagnosis^25^ is one useful tool to address how trade-offs in testing should be managed, but will require concerted epidemiological research, and agreement on how to decide when the trade-off are permissible.

#### Patient preferences and epistemic injustice

We observed that debates about testing appear to be happening chiefly amongst clinicians, who conduct a risk analysis such as above, and decide whether a test is indicated. However, there are sometimes instances when different stakeholders may make different judgements about whether trade-offs are permissible. One well known historical example of this phenomenon is that of natalizumab, a medication for the treatment of multiple sclerosis: natalizumab was initially withdrawn from market due to concern by the manufacturer that an associated risk of a serious adverse event (progressive multifocal leukoencephalopathy) was an impermissible trade-off despite its good efficacy, yet was returned to market a year later in part due to patient advocacy^26^. It may be noted that one reasonable view, grounded in considerations of respect for patient autonomy, would therefore be that decisions on whether to pursue a test should consider the preferences and values of patients and families in more detail. Clinicians would best respect autonomy by incorporating these preferences rather than deciding for patients based on implicit judgments of beneficence masquerading as whether a test is “medically indicated” or not. There is indeed good literature suggesting that many physicians can be influenced by medically irrelevant features when deciding whether ‘borderline’ cases have a medical indication^27^. Yet, where there is room for reasonable disagreement about which course of action to take, either because of empirical uncertainty or because there are competing values at stake, considerations of autonomy have a particular ethical significance.

A more patient-centered approach would be to extend the borderline or grey zone where decisions should refer to the patient’s own values. As one of us has argued, we should defer to patients or their surrogate decision makers in cases where there is reasonable dissensus, that is, where some reasonable physicians would support the patient or parent’s choice, even if there is general consensus against it^28, 29^.

The principle of respect for autonomy thus lends support to the practice of ascertaining patient preferences in these complex cases. However, such practices also receive support from considerations of justice. Indeed, a failure to accommodate the views of patients can plausibly serve to exacerbate the epistemic injustice that psychiatric patients commonly face, i.e. the inappropriate discounting of the knowledge or preferences of the patient^30^. Epistemic injustice is “a harm done to a person in her capacity as an epistemic subject (a knower, a reasoner, a questioner)”.^30^ This might take the form of ‘testimonial injustice’, where negative prejudice or stigma causes others to diminish the credibility assigned to the individual’s testimony.^31^ This might include testimony about their symptoms and their own views about what is in their interests - yet failing to seek the patient’s testimony in the first place can also be understood to constitute epistemic injustice. Accordingly, further research into the lived experience of individuals involved in these complex cases is crucial to ensure that the profession can mitigate the potential for epistemic injustice in developing an ethically sound clinical approach to these cases. To amplify the significance of this research, systematic data should be collected to evidence the epistemic and ethical impacts of lived experience contributions^32^

## Summary and Limitations

Most of the ethical challenges on the use of advanced diagnostics for rare, identifiable causes of psychosis we have discussed coalesce around i) inequities to access to invasive, or advanced diagnostics necessary for the workup of identifiable causes of psychosis, ii) responsibilities around those inequities, including who and what systems should address these inequities, and iii) the risk that pursuing a comprehensive workup can sometimes worsen existing biases that further limit access to good mental health care. Therefore, we might suggest several recommended focus areas for future empirical work: 1) *how big* are inequities in access and *how harmful are they*, that is, to what extent do they worsen health disparities for those with psychiatric symptoms? 2) what are the systems factors that affect the ability, or inability to appropriately assess for identifiable causes of psychosis for example, how important is the assembling of interdisciplinary medical teams? 3) what are the views of people with lived experience of psychosis and family members on these issues and priorities for future work? Is there evidence that integrating lived experience priorities positively impacts inequities in access to mental health care for this population? Some of the concerns for injustice can be about whose voices are not ‘at the table’ in decisions about investigations for rare causes of psychosis.

We anticipate that some may argue that responsibility for this work on identifiable causes of psychosis is beyond the remit and responsibility of psychiatry. We would suggest that psychiatric symptoms due to identifiable (and sometimes reversible) biological causes, whilst they have been increasingly excluded from the remit of psychiatry in the last century, should not be eschewed in this century. Psychiatry will often be an early point of contact for patients presenting with psychosis, and whether or not the underlying causes for the psychosis are known does not change the fact that the symptoms are psychiatric. Interdisciplinary teams are essential, and it is imperative that psychiatry become comfortable with advances in diagnostics tests available to exclude underlying medical cases.

Finally, we acknowledge that there are additional ethical challenges; the ethical issues highlighted here are certainly influenced by the particularities of the cases we as a group have experienced and considered most closely, and thus, discussion of other cases would be rightly hypothesized to highlight other issues. For example, one limitation of our perspective is that we have focused largely on fluid, imaging, or electrophysiological biomarkers, and there are some special considerations when it comes to genetic tests (whole genome sequencing, whole exome sequencing, microarrays), which we have not discussed here. For example, genetic testing can reveal information not just about the patient, but also about family members or future children. Moreover, in many jurisdictions there are special laws regarding genetic information, for example, the Genetic Information Nondiscrimination Act (GINA) in the united states, which protects individuals from discrimination based on genetic information but does not apply to other types of tests^12^. Additionally, We have not considered ethical issues related to the trajectory of expanding knowledge of rare disease; for example, it was recently demonstrated that a notable minority of people enrolled in clinical trials and diagnosed as having common diseases have rare mendelian genetic conditions discoverable by genome sequencing that are likely responsible for their symptoms and different responses to medications^33^. As such, it is possible that common diagnoses in psychiatry may increasingly be revealed to contain collections of rare disorders with overlapping phenotypes.

In conclusion, we hope that the model for interdisciplinary perspective here might inspire further discussion at institutions grappling with these critical issues.

## Box 1

Search of PubMed for literature on the ethical challenges of diagnostic technologies for rare causes of psychosis yields zero results

*PubMed Search:* the search of PubMed was conducted December 2024 using two common terms for rare identifiable causes of psychosis 1) “secondary psychosis” or 2) “organic psychosis” AND “ethics” or “ethical”.

This perspective piece is motivated by clinical cases we have encountered or discussed, such as the generalized anonymous case in Box 2, where there is a perceived ethical question around diagnostic investigation - both fluid biosamples (e.g. lumbar puncture [LP] and cerebrospinal fluid [CSF] assays, etc.) or neuroimaging approaches.

## Box 2

The type of clinical case that raises key ethical issues and relevant future directions on use of novel diagnostics to determine rare identifiable causes of psychosis

Case: the case below is generalized and anonymous, not reflecting any particular case):

*A young woman with no relevant past medical history is in her usual state of health until she develops sleep disturbance including nightmares and insomnia. Her friends and family become concerned when she speaks about increasing paranoid thoughts and hearing sounds that are not there. She is referred to an outpatient first episode psychosis clinic where her symptoms are treated with benzodiazepines and antipsychotic medications. However, she rapidly deteriorates, with unpredictable behavior and psychomotor agitation. She is brought into hospital and admitted to an inpatient psychiatry ward. There, it is noticed she is posturing and exhibiting odd motor movements raising concern around catatonia. Given the movement disorder and the location of the unit within a general hospital with comprehensive subspecialty consult services readily available, the team consults neurology. Neurology raises concern for autoimmune encephalitis, which is confirmed with LP and CSF testing that detects anti-neuronal autoantibodies. She is treated with high dose steroids and IVIg, which leads to rapid improvement of her symptoms. She is able to discontinue antipsychotic medications and begin a slow taper of benzodiazepines*.

Questions:

- What would have happened if the primary team were not as familiar with red flags for secondary psychoses?
- How easily could this case have been missed or diagnosis delayed? What were the systems and individual factors involved?
- What if the patient was at a hospital where neurological consultation was unavailable or that did not have the capability to perform LP and autoantibody testing?
